# A novel hemagglutinin protein produced in bacteria protects chickens against H5N1 highly pathogenic avian influenza viruses by inducing H5 subtype-specific neutralizing antibodies

**DOI:** 10.1371/journal.pone.0172008

**Published:** 2017-02-17

**Authors:** Violetta Sączyńska, Agnieszka Romanik, Katarzyna Florys, Violetta Cecuda-Adamczewska, Małgorzata Kęsik-Brodacka, Krzysztof Śmietanka, Monika Olszewska, Katarzyna Domańska-Blicharz, Zenon Minta, Bogusław Szewczyk, Grażyna Płucienniczak, Andrzej Płucienniczak

**Affiliations:** 1 Institute of Biotechnology and Antibiotics, Warsaw, Poland; 2 Department of Poultry Diseases, National Veterinary Research Institute, Puławy, Poland; 3 Department of Recombinant Vaccines, Intercollegiate Faculty of Biotechnology, University of Gdańsk and Medical University of Gdańsk, Gdańsk, Poland; University of South Dakota, UNITED STATES

## Abstract

The highly pathogenic (HP) H5N1 avian influenza viruses (AIVs) cause a mortality rate of up to 100% in infected chickens and pose a permanent pandemic threat. Attempts to obtain effective vaccines against H5N1 HPAIVs have focused on hemagglutinin (HA), an immunodominant viral antigen capable of eliciting neutralizing antibodies. The vast majority of vaccine projects have been performed using eukaryotic expression systems. In contrast, we used a bacterial expression system to produce vaccine HA protein (bacterial HA) according to our own design. The HA protein with the sequence of the H5N1 HPAIV strain was efficiently expressed in *Escherichia coli*, recovered in the form of inclusion bodies and refolded by dilution between two chromatographic purification steps. Antigenicity studies showed that the resulting antigen, referred to as rH5-*E*. *coli*, preserves conformational epitopes targeted by antibodies specific for H5-subtype HAs, inhibiting hemagglutination and/or neutralizing influenza viruses *in vitro*. The proper conformation of this protein and its ability to form functional oligomers were confirmed by a hemagglutination test. Consistent with the biochemical characteristics, prime-boost immunizations with adjuvanted rH5-*E*. *coli* protected 100% and 70% of specific pathogen-free, layer-type chickens against challenge with homologous and heterologous H5N1 HPAIVs, respectively. The observed protection was related to the positivity in the FluAC H5 test (IDVet) but not to hemagglutination-inhibiting antibody titers. Due to full protection, the effective contact transmission of the homologous challenge virus did not occur. Survivors from both challenges did not or only transiently shed the viruses, as established by viral RNA detection in oropharyngeal and cloacal swabs. Our results demonstrate that vaccination with rH5-*E*. *coli* could confer control of H5N1 HPAIV infection and transmission rates in chicken flocks, accompanied by reduced virus shedding. Moreover, the role of H5 subtype-specific neutralizing antibodies in anti-influenza immunity and a novel correlate of protection are indicated.

## Introduction

Influenza viruses (IVs) belong to the *Orthomyxoviridae* family, which consists of six genera [1]. Strains of the most epidemiologically relevant *Influenza virus A* genus are further classified by reference to the subtypes of hemagglutinin (HA) and neuraminidase (NA), surface glycoproteins. In the major reservoir of wild aquatic birds, Influenza A viruses exist as various combinations of H1-H16 HAs and N1-N9 NAs. It is widely recognized that Avian IVs (AIVs) of the H5 and H7 subtypes, usually non-pathogenic in their natural waterfowl hosts, may become highly pathogenic (HP) once introduced into a susceptible poultry population. This was the case of H5N1 HPAIV, detected for the first time in China among farmed geese in 1996 and in humans a year later [2]. Since then, H5N1 HPAIVs have spread to many regions of the world [3]. This spreading was accompanied by frequent avian flu outbreaks in poultry, resulting in a mortality rate of up to 100%. Moreover, until February 2016, there have been a total of 846 laboratory-confirmed human cases of H5N1 influenza, 449 of which had a mortal outcome [4]. During the initial circulation and spread of the H5N1 viruses, the HA genes diversified into multiple genetic lineages, termed clades [3]. From 2009 onward, the emergence of reassortant H5-subtype HPAIVs, such as H5N2, H5N5, H5N6 and H5N8, has been noted. The novel H5N8 and H5N2 HPAIVs, identified in 2014, spread rapidly and substantially affected many populations of domestic birds due to infection or mass culling [5,6]. Thus, there is still a risk that circulating H5N1 HPAIVs will give rise to new dangerous reassortants or acquire the ability to transmit directly between humans. Taken together, it is of veterinary and public health significance to develop effective vaccines against H5N1 HPAIVs. Such vaccines can be produced in a relatively short time, contrary to the traditional vaccines that are grown in chicken eggs or cell cultures.

The demand for the efficient production of vaccines against influenza, especially in case of a pandemic threat, could be met using recombinant DNA technology for subunit vaccine manufacturing. An important advantage of such vaccines is that their usage allows for the serological differentiation of naturally infected animals/flocks from vaccinated ones, the so-called DIVA strategy, which is a prerequisite for vaccinations against HPAIVs [7]. The obvious candidate for the production of subunit vaccines against flu is HA, a key viral protein capable of eliciting potent neutralizing antibodies. To obtain HA antigens of various subtypes, including H5, yeast (e.g., [8]), baculovirus (e.g., [9]) and mammalian (e.g., [10]) expression systems, but not a bacterial expression system, have been used for years. The exploitation of bacteria enabling the relatively easy, low-cost and efficient production of the vaccine protein seems to have been hampered for a long time by the commonly accepted view that glycosylation determines the correct HA structure. More recently, experimental data for both the considerable independence of HA folding from the glycosylation status and the successful production of HA proteins in bacteria (bacterial HAs) have been published (for review, see [11]). In contrast to full-length or ectodomain-based HAs produced in eukaryotic expression systems, the vast majority of antigens overexpressed in bacteria are based on the HA1 subunit, and only a few examples of bacterial HAs based on the ectodomain or the HA2 subunit have been reported so far [11].

In this paper, we describe for the first time the ectodomain-based HA protein produced in a bacterial expression system. The HA protein (aa 17–522, ΔRRRKKR) with the sequence of the H5N1 HP viral strain was efficiently expressed in *Escherichia coli*, refolded and chromatographically purified from inclusion bodies (IBs). The antigen, referred to as rH5-*E*. *coli*, has been studied for antigenicity, the ability to bind to sialic acid-containing receptors and oligomerization status. Finally, the immunogenicity and efficacy of rH5-*E*. *coli* were verified by means of homologous and heterologous challenge experiments. The vaccine potential of the novel HA protein produced in bacteria was confirmed. Moreover, serological analyses demonstrated the role of H5 subtype-specific, non-hemagglutination-inhibiting neutralizing antibodies in conferring anti-influenza virus immunity and providing a new immunologic correlate of protection for anti-H5N1 HPAIV vaccines.

## Materials and methods

### Genetic construction

The full-length cDNA encoding HA was obtained by reverse transcription and subsequent amplification (RT-PCR) using an RNA template originating from the H5N1 A/swan/Poland 305-135V08-2006 strain of HPAIV (EpiFlu Database [http://platform.gisaid.org]; Accession No. EPI156789). This was the initial step in obtaining a cDNA clone encoding a vaccine antigen expressed in bacteria. The target antigen was the ectodomain-based HA protein (aa 17–522) with a deletion of RRRKKR (Δ341–346), the proteolytic cleavage site. The protein terminated after the bromelain cleavage site (aa 521–522).

Briefly, the pBluescriptHA plasmid, which carries the codon-optimized gene encoding HA, was the template for the DNA fragment corresponding to amino acids 17–522. The fragment was amplified using primers introducing NdeI and XhoI restriction sites at the 5’ and 3’ ends, respectively. The obtained fragment was inserted into an NdeI- and XhoI-digested pIGCmT7Kes expression plasmid [12] and transformed into the NM522 *E*. *coli* strain. Subsequently, the plasmid was used as a template to amplify a DNA fragment encoding HA without the sequence encoding amino acids 341–346 (RRRKKR). The amplified and digested fragment was inserted into the pIGCmT7Kes plasmid, and the resulting pIGKesHA17522Δ plasmid was transformed into the NM522 *E*. *coli* strain for cloning. All the generated constructs were verified by DNA sequencing. The pIGKesHA17522Δ plasmid was transformed into the BL21 (DE3) *E*. *coli* strain for expression. Glycerol stocks of the transformed BL21 (DE3) *E*. *coli* strain were stored at -70°C.

### Protein expression, refolding and purification

*E*. *coli* BL21 (DE3) cells harboring the recombinant plasmid were cultured in shaking flasks at 150 rpm and 25°C using LB medium with chloramphenicol (12 μg/mL) until reaching an OD600 of ~0.6. Protein expression was induced by adding IPTG to a final concentration of 0.1 μg/mL. Bacteria were grown for the next 4½ h under the conditions stated above, harvested by centrifugation and resuspended in a lysis buffer (0.05 M Tris-HCl, 0.5 M NaCl, 0.01 M EDTA, 0.005 M 2-mercaptoethanol, 0.35 mg/mL lysozyme, 1% PMSF, pH 7.5). After the 30-minute incubation at 20°C, Triton X-100 was added to a concentration of 1%, and the suspension was sonicated and centrifuged. The obtained pellet was processed by successive suspending and centrifugation using PBS buffer containing 1% Triton X-100 or 2 M urea. The isolated inclusion bodies (IBs) were washed twice using PBS and then aliquoted and stored at -20°C until further use.

To follow the procedure, the final IBs were solubilized in the inclusion body dissolution buffer (50 mM Tris-HCl, 8 M urea, 10 mM 2-mercaptoethanol, 0.01% Triton X-100, pH 8.0) for 1–2 h at room temperature and then centrifuged and filtered through 0.2-μm filters. The resulting protein solution was loaded onto a DEAE Sepharose Fast Flow column (GE Healthcare, Sweden) that had been pre-equilibrated with a calibration buffer (50 mM Tris-HCl, 6 M urea, 0.1% Triton X-100, pH 8.0). After the column was washed with the calibration buffer, the bound proteins were eluted with an elution buffer (50 mM Tris-HCl, 6 M urea, 800 mM NaCl, pH 8.0), and peak fractions containing the HA protein were pooled. Subsequently, the collected proteins were diluted to 0.07–0.09 mg/mL in 40 mM Tris-HCl buffer containing 100 mM NaCl (final pH 8.0) and then allowed to refold for the next 15–16 h at 4°C with mixing. After filtering through 0.4-μm filters, refolded proteins were applied to a Phenyl Sepharose 6 Fast Flow (High-Sub) column (GE Healthcare, Sweden) that had been pre-equilibrated with a calibration buffer (40 mM Tris-HCl, 100 mM NaCl, pH 8.0). Unbound proteins were washed out with the calibration buffer, while the bound proteins were eluted with deionized water. After the addition of 1 M Tris-HCl buffer, pH 8.0, to a final concentration of 40 mM and a protease inhibitor cocktail (Sigma-Aldrich, USA) to a concentration recommended by the manufacturer, the pooled fractions of HA protein were filtered through 0.2-μm filters and stored in aliquots at 4°C. The protein obtained in this way was referred to as rH5-*E*. *coli*.

General-purpose laboratory reagents that were used to produce rH5-*E*. *coli* were purchased from Sigma-Aldrich (USA). The protein concentration in the samples collected at successive steps of the procedure was determined by the Bradford method. The expression of the HA protein and its purification were followed by sodium dodecyl sulfate-polyacrylamide gel electrophoresis (SDS-PAGE). The quantitative composition of the final rH5-*E*. *coli* preparations was determined by on-chip separation and detection in an Agilent 2100 Bioanalyzer (Agilent Technologies, USA) using the Agilent High Sensitivity Protein 250 kit. In this way, the purity of different HA protein batches was estimated from 75% to 80%. The exact mass of the rH5-*E*. *coli* was determined with the use of Matrix Assisted Laser Desorption Ionization Time of Flight (MALDI-TOF/TOF) MS instrument (4800 Plus, AB SCIEX, USA). For comparison, the molecular mass was calculated from the amino acid composition using GPMAW 8.2 software (Lighthouse, Denmark). The antigenicity of rH5-*E*. *coli* was studied by ELISA using commercial monoclonal and polyclonal antibodies (mAbs and pAbs, respectively) against H5 HA, listed in Table A in S1 Appendix. The sialic acid-binding activity and the oligomerization status of rH5-*E*. *coli* were assessed using a hemagglutination test. Details on the SDS-PAGE analysis and the ELISA and hemagglutination tests are provided in S1 Appendix.

### Influenza viruses and stock preparation

The highly pathogenic AIVs used in this work were propagated in the allantoic cavities of embryonated chicken eggs (Valo-Biomedia, Germany) under biosafety level 3 conditions at the National Veterinary Research Institute (Pulawy, Poland). They were stored in aliquots at -70°C (for challenge) or inactivated with 0.1% formaldehyde (Sigma-Aldrich, USA) for 2 h at 37°C (for the hemagglutination inhibition test). The viral stocks that were stored at -70°C were titrated before use.

### Vaccination, challenge and sample collection

The challenge experiments were conducted in laying-type, White Leghorn, specific pathogen-free (SPF) chickens in a biosafety level 3 containment at the National Veterinary Research Institute (Pulawy, Poland). The scheme of the studies was in accordance with the requirements of the World Organization for Animal Health (OIE) [13]. These requirements included a challenge HPAIV dose and time interval between vaccination and exposure to challenge viruses.

A group of 3- (Exp 1) and 3½- (Exp 2) week-old SPF chickens (10 birds/group) were immunized subcutaneously twice at a 4-week interval with 25 μg of rH5-*E*. *coli* and aluminum hydroxide adjuvant (1.3% Alhydrogel, Brenntag Biosector, Denmark). During the time-course of vaccinations, blood samples for serological analyses were collected. Three weeks after the second dose was administered, the chickens were inoculated intranasally/intraocularly (in/io) with a 10^6^ median egg infectious dose (EID_50_) of HPAIVs in a volume of 100 μL. Homologous HPAIV—A/turkey/Poland/35/07(H5N1) from clade 2.2 and heterologous HPAIV—A/crested eagle/Belgium/01/2004(H5N1) from clade 1 were the infectious viruses in Experiments 1 and 2, respectively. The controls for the challenge experiments included non-vaccinated contact (CC) chickens that were introduced to the vaccinated groups 24 h after infection (2 birds/group), as well as non-vaccinated and infected (C) animals (5 birds/group).

At 14 days post-infection (dpi), clinical observations of the animals and sample collection were performed. For virus detection, oropharyngeal and cloacal swabs were collected at different dpi or postmortem (pm). In addition, organs (brain, lungs, kidney, spleen) were taken from dead chickens to determine viral RNA titers. Blood samples for serological analyses were collected from all of the survivors at 14 dpi.

### Serological analyses

Serum samples that were collected during the challenge experiments were analyzed using the hemagglutination inhibition (HI) test and two commercial AIV antibody ELISA kits. The HI test was conducted according to the OIE Manual of Diagnostic Tests and Vaccines for Terrestrial Animals [13]. Chicken sera from both experiments were tested with homologous H5N1 HPAIV at a hemagglutination inhibition unit (HIU) of 1:8 using SPF chicken erythrocytes. The HI titer was determined as the reciprocal of the highest dilution of serum that caused an inhibition of hemagglutination activity with 4 hemagglutination units (HAU) of the inactivated antigen. Serum HI titers equal to or greater than 1:16 were considered positive. For test vaccine groups, the Geometric Mean Titers (GMT) of HI-positive sera at each sampling time point were calculated.

Avian Influenza Virus Antibody Test Kit, ELISA—AI MultiS-Screen (Idexx Laboratories, Inc., USA) and ID Screen Influenza H5 Antibody Competition—FluAC H5 (IDVet, France) detect antibodies against nucleoprotein (NP) and H5 HA of influenza A viruses in bird sera, respectively. The AI MultiS-Screen test was performed according to the manufacturer’s instructions. In the FluAC H5 test, the basic protocol was followed (serum samples at a 1:5 dilution were incubated on plates for 1 h at 37°C). In both analyses, the results-to-negative-control absorbance ratio of a test sample was calculated and expressed as an S/N ratio (AI MultiS-Screen test) or a competition percentage (FluAC H5 test). Samples presenting a competition percentage ≥40%, between 35% and 40% or ≤35% were considered negative, doubtful or positive for the presence of anti-H5 HA antibodies, respectively. Samples with an S/N ratio ≥0.5 were considered negative, and those with an S/N ratio <0.5 were considered positive for the presence of anti-NP antibodies. For test vaccine groups at each sampling time point, the mean values of an S/N ratio and the competition percentage in the AI MultiS-Screen and FluAC H5 tests were calculated, respectively.

### Viral RNA determinations

Oropharyngeal, cloacal swabs and organs (brain, lungs, kidney, spleen), collected after challenging the chickens, were stored in a universal transport medium (COPAN Diagnostics, Inc., Italy) until viral RNA analyses. Samples from individual chickens were analyzed separately, except for organs from positive controls of the heterologous challenge experiment (Exp 2), which were determined after pooling. Total RNA was extracted from 0.1 mL of medium using the RNeasy Mini Kit (Qiagen, Germany). Quantitative real-time mRT-PCR was performed as described by Spackman et al. [14]. The oligonucleotides M-25 (5’-AGATGAGTCTTCTAACCGAGGTCG-3’) and M-124 (5’-TGCAAAAACATCTTCAAGTCTCT-3’) were used as primers, while M-64 (5’-FAM-TCAGGCCCCCTCAAAGCCGATAMRA-3’) served as a probe. Quantitative RNA standards with known virus titers, extracted from 10-fold dilutions of challenge viruses, were used to convert the Ct values of RT-qPCR to the egg infectious dose equivalent values (eqEID_50_) *per* milliliter of swab fluid or gram of tissue. The amounts of viral RNA in the tested samples were extrapolated from the standard curves and expressed as log_10_ eqEID_50_ *per* milliliter (swabs) or gram (tissue). Based on these determinations, the GMT of viral RNA for virus-positive samples collected at subsequent dpi or pm in individual chicken groups was calculated.

### Ethic statement

The challenge experiments in chickens were approved by the Second Local Ethical Committee for Animal Experiments at the University of Life Sciences in Lublin (Poland), Permit Number 26/2012. All efforts were made to minimize the suffering of the animals. The chickens were monitored twice a day (morning and afternoon), including weekends. The humane endpoints for early termination of the experiment were defined in advance and based on the following clinical criteria: a) severe prostration, b) severe dyspnea, c) lethargy, d) severe nervous symptoms, including opistothonus, torticollis, trembling and paralysis. However, due to the very severe and violent clinical course of the highly pathogenic avian influenza H5N1 in chickens, the birds which died were always found dead in the morning despite the fact that in the previous afternoon they were clinically healthy or displayed only mild clinical symptoms (apathy). It is very common that the health status in the course of H5N1 infection in gallinaceous poultry is deteriorating very quickly and death occurs within a few hours. The use of euthanasia during experiment certainly would influence on evaluation of viral replication and dissemination in infected animals, contact transmission and shedding of the challenge viruses, thereby on the final conclusions. Therefore, we did not use humane endpoints during the animal survival study. At the end of the experiment the chickens were humanely euthanized by cervical dislocation.

## Results

### Vaccine antigen (rH5-*E*. *coli*)

The antigen produced in bacteria for vaccination against the flu was designed as the ectodomain-based, soluble HA protein of H5N1 HPAIV with a polybasic cleavage site deletion. Consistently, the designed protein contained the full-length HA1 subunit encompassing the viral HA globular domain with the host cell receptor-binding site. It also contained a fragment of the stalk domain-forming HA2 subunit with a fusion peptide and bromelain cleavage site at N- and C-termini, respectively. The target protein was deprived of the signal peptide, characteristic only of the precursor form of the antigen, as well as the transmembrane and cytoplasmic domains of mature viral HA. The antigen was designed using an HA sequence from the H5N1 A/swan/Poland 305-135V08-2006 strain of HPAIV [15], depicted in Table 1.

**Table 1:** Influenza viruses used in this work.

Application	Relevant HPAIV strain	Clade	Origin
Source sequence for rH5-E. coli	A/swan/Poland/305-135V08/2006(H5N1)	2.2	Department of Poultry Diseases, National Veterinary Research Institute, Pulawy, Poland
Infection in experiment 1	A/turkey/Poland/35/07(H5N1)	2.2	Department of Poultry Diseases, National Veterinary Research Institute, Pulawy, Poland
Infection in experiment 2	A/crested eagle/Belgium/01/2004(H5N1)	1	Dr. T. van den Berg CODA-CERVA, Brussels, Belgium
Hemagglutination inhibition (HI) test	A/turkey/Poland/35/07(H5N1)	2.2	Department of Poultry Diseases, National Veterinary Research Institute, Pulawy, Poland

The HA protein (aa 17–522, ΔRRRKKR) was efficiently expressed in the *E*. *coli* BL21(DE3) strain transformed with the pIGCmT7Kes plasmid [12] harboring the relevant codon-optimized DNA fragment. Because the target antigen has been recovered in the form of IBs, the procedure for obtaining the antigen required the solubilization of isolated IBs in denaturing buffer and HA protein refolding in addition to the purification step. During the development of a quality-focused procedure for the laboratory-scale production, the purification progress was examined mainly by SDS-PAGE and the HA antigenicity by ELISA. The final protocol included the isolation of IBs and, their solubilization in the urea-based dissolution buffer. The protein yields were 67–87 mg/L culture. The next stage was protein purification on a DEAE-Sepharose bed. The partially purified protein was refolded by dilution and thereafter subjected to purification on a Phenyl-Sepharose bed. The resulting protein preparation was formulated in 40 mM Tris-HCl buffer, pH 8.0, with the addition of protease inhibitors. HA antigen obtained at the laboratory scale was referred as rH5-*E*. *coli*. The purity of rH5-*E*. *coli* typically ranged from 75% to 80%, as estimated by on-chip separation and detection in the Agilent 2100 Bioanalyzer. The antigen yields were 1–2 mg/L culture. More information on the process of the rH5-*E*. *coli* production is provided in S1 Appendix.

Prior to use in the vaccine formulation, rH5-*E*. *coli* was characterized by mass spectrometry, ELISA for antigenicity measurement and a hemagglutination test. Protein analyses were performed in parallel with the ectodomain-based H5 HA proteins (aa 17–530, ΔRRRKKR, 6x His) produced using a mammalian-cell expression system (rH5-mammalian) and a baculovirus expression vector system (rH5-BEVS). The rH5-mammalian was highly homologous with both rH5-BEVS and rH5-*E*. *coli*, which originated from the same strain of H5N1 HPAIV (Table 1). The BLAST algorithm found that rH5-mammalian and rH5-*E*. *coli* share 99% identity and 100% positivity of amino acid 17–522 sequences. The reference antigens are described in details in S1 Appendix. A molecular mass of ~57 kDa for bacterial HA was determined using MALDI-TOF/TOF MS. This value is consistent with that calculated from the amino acid composition and contrasts with the determined masses of rH5-mammalian (76 kDa) and rH5-BEVS (64 kDa), which differed from the theoretical values by ~18 kDa and ~6 kDa, respectively, due to glycosylation.

The antigenicity of rH5-*E*. *coli* was studied by ELISA using commercial monoclonal and polyclonal antibodies (mAbs and pAbs, respectively) against H5 HA, as listed in Table A in S1 Appendix. Most of the mAbs, denoted mAb 1 to mAb 9, were specified as displaying characteristics of H5 subtype-specific Abs (6/9) and recognizing H5 HAs in the hemagglutination inhibition (HI) test (8/9). One antibody clone was characterized as active in both the HI and virus neutralization (VN) tests. Two pAb items that were used in this work (pAb 1 and pAb 2) differed in that pAb 1 was directed against HA1 and pAb 2 against HA2 of H5 HA. The utility of the selected Abs for analyses of rH5-*E*. *coli* was further evaluated by testing their reactivity with commercially available H5 HA proteins. Detailed data on mAb 1 to mAb 9, pAb 1 and pAb 2 from previous specifications and our study are provided in S1 Appendix. ELISA with mAbs and pAbs (Table A in S1 Appendix) allowed us to discriminate between properly folded and misfolded HA antigens. Used for antigenicity studies, the assay showed that rH5-*E*. *coli* is recognized by all of the used antibodies, similar to reference H5 HA antigens (Fig B in S1 Appendix). At the applied antigen and antibody concentrations, the recognition profiles of rH5-*E*. *coli* and rH5-mammalian by mAbs and pAbs were essentially indistinguishable, and both differed from that of rH5-BEVS by having a ~2-fold higher pAb 2 reactivity. Altogether, these results indicate that rH5-*E*. *coli* contains well-preserved conformational epitopes of native HA, which are essential for the induction of the protective immune responses. The produced antigen adopted proper conformation even though it was not glycosylated and was devoid of the regions known to participate in HA folding during viral multiplication.

The binding to sialic acid-containing receptors and the oligomerization status of rH5-*E*. *coli* were assessed by a hemagglutination test using red blood cells (RBCs) from specific pathogen-free (SPF) chickens. It has been shown that rH5-*E*. *coli* can bind and agglutinate RBCs, in contrast to its 2-mercaptoethanol-reduced form (Table B in S1 Appendix). This indicates that the receptor binding and oligomeric HA activities of viral antigen were preserved. Hemagglutination was observed at a minimum rH5-*E*. *coli* amount of 0.5 μg. For comparison, more rH5-BEVS but less rH5-mammalian was required to evoke hemagglutination. Thus, rH5-*E*. *coli* was found to exist in part as a functional oligomer, which is an advantageous characteristic of a vaccine HA.

### Mortality and morbidity in challenge experiments

**Fig 1:**
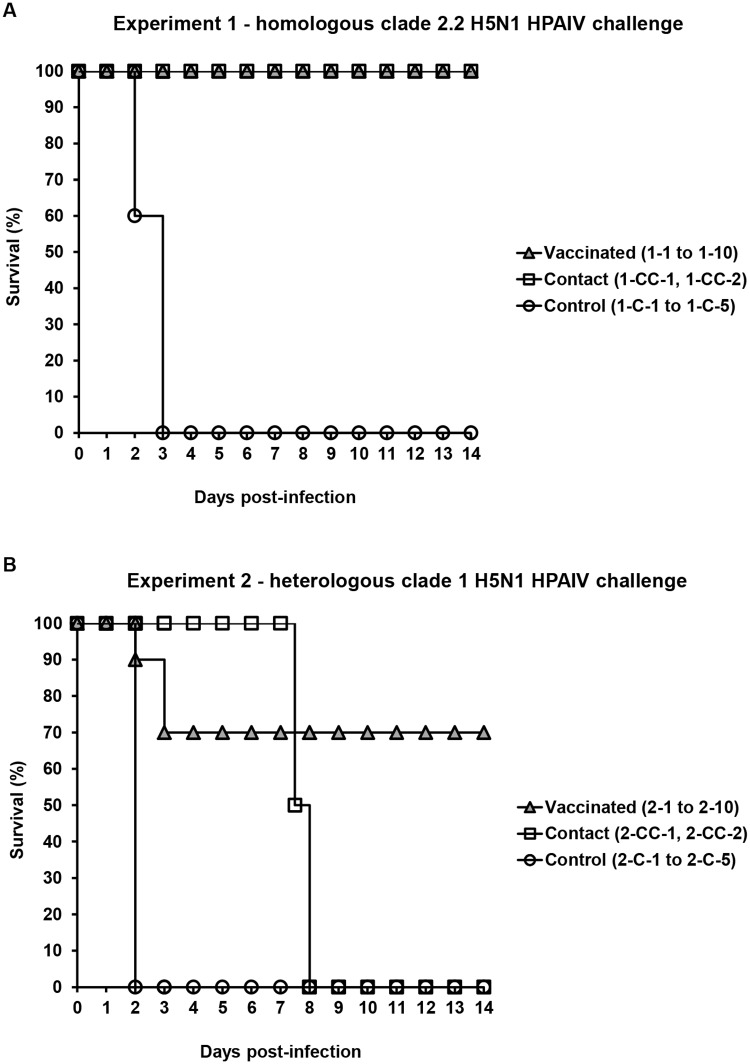
Survival rates in the challenge experiments. A group of 3-week-old specific pathogen-free (SPF) chickens denoted 1–1 to 1–10 (Exp 1) and 3½-week-old ones denoted 2–1 to 2–10 (Exp 2) were vaccinated subcutaneously twice at a 4-week interval with 25 μg of rH5-*E*. *coli* and aluminum hydroxide adjuvant (Alhydrogel). Three weeks after the boosts, the chickens were inoculated intranasally/intraocularly (in/io) with 10^6^ 50% egg infectious doses (EID_50_) of (A) clade 2.2 homologous (Exp 1) or (B) clade 1 heterologous (Exp 2) H5N1 HPAIVs, as depicted in Table 1. Approximately 24 hours after inoculation, non-vaccinated contact SPF chickens denoted 1-CC-1, 1-CC-2 (Exp 1) and 2-CC-1, 2-CC-2 (Exp 2) were introduced to the tested groups. Untreated, fully susceptible SPF chickens denoted 1-C-1 to 1-C-5 (Exp 1) and 2-C-1 to 2-C-5 (Exp 2) that were infected in/io with challenge viruses at the same age as the vaccinated animals served as positive controls. The data are presented as the survival percentage in the respective groups on each day during the 2-week observation period.

rH5-*E*. *coli* was examined for vaccination efficacy by homologous and heterologous challenge experiments (Exp 1 and Exp 2, respectively) in SPF laying-type chickens. The A/turkey/Poland/35/07(H5N1) strain from clade 2.2 and the A/crested eagle/Belgium/01/2004(H5N1) strain from clade 1 were the infectious viruses used for the challenge experiments, as depicted in Table 1. These experiments were performed on two groups of chickens, each containing ten animals. Vaccination was performed twice at a 4-week interval by the subcutaneous administration of 25 μg of rH5-*E*. *coli* adjuvanted with aluminum hydroxide. Three weeks after booster immunizations, the birds were inoculated intranasally/intraocularly (in/io) with 10^6^ 50% egg infectious doses (EID_50_) of H5N1 HPAIVs. Approximately 24 hours after infection, two non-vaccinated contact chickens were introduced to each test group to monitor virus transmission. The positive controls in these experiments were untreated, fully susceptible chickens, five in each group, that were inoculated in/io with H5N1 HPAIVs at the same doses as vaccinated animals. Clinical observations of the birds were performed for 14 days post-infection (dpi). The survival rates of vaccinated, contact and control chickens in the homologous and heterologous challenge experiments are presented in Fig 1A and 1B, respectively.

All of the chickens that were vaccinated with adjuvanted rH5-*E*. *coli* and then challenged with the homologous H5N1 HPAIV, as well as both contact chickens, were alive and did not exhibit any clinical symptoms of influenza (Fig 1A). In contrast, two chickens from the positive control group died on day 2 and the other three on day 3 after infection with this virus. In the heterologous challenge experiment, seven out of ten vaccinated chickens survived (Fig 1B). Four of them had no signs of influenza morbidity. The remaining three animals got sick from the flu but recovered and did not show any disease symptoms at 14 dpi. One vaccinated chicken of Exp 2 died on day 2 and two others on day 3 after heterologous H5N1 virus challenge. In this experiment, both contact chickens were alive longer than expected, i.e., until day 7 after the vaccinated birds had been challenged. One of them died between 7 and 8 dpi and the second at 8 dpi. Control group animals quickly succumbed to virus infection and died at 2 dpi. To summarize, the administration of two rH5-*E*. *coli* doses in the presence of aluminum hydroxide adjuvant protected 100% of the SPF, layer-type chickens against challenge with the homologous H5N1 HPAIV, while 70% were protected against infection with the heterologous H5N1 HPAIV (Fig 1A and 1B). Moreover, chicken vaccination eliminated or delayed the contact transmission of homologous and heterologous challenge viruses, respectively.

### Chicken humoral responses

**Fig 2:**
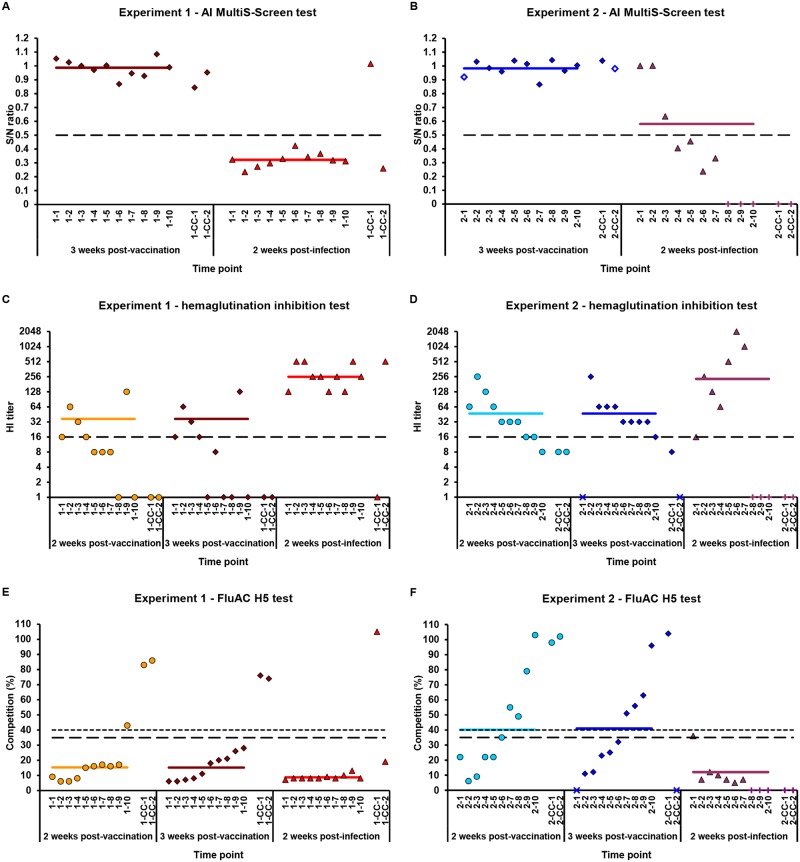
Chicken immune responses in the course of the challenge experiments. Chickens were vaccinated and challenged as described in Fig 1. Antibodies to influenza A virus antigens in chicken sera from (A, C, E) homologous (Exp 1) and (B, D, F) heterologous (Exp 2) challenge experiments were measured by (A-B) AI MultiS-Screen (Idexx Lab), (C-D) hemagglutination inhibition (HI) and (E-F) FluAC H5 (IDVet) tests. These tests were conducted and interpreted as described in the Materials and Methods. The levels of antibodies against (A-B) nucleoprotein (NP) and (C-D) H5 HA in the test samples (S) were evaluated in relation to the negative controls (N) and are presented as an S/N ratio and a competition percentage, respectively. The HI activities of sera towards homologous H5N1 virus (A-B) are displayed typically as HI antibody titers. Only the results for vaccinated and contact chickens are presented. Positive controls were seronegative in the respective tests before infection and were not examined after challenge due to death. Data are shown for individual chickens at 2 and/or 3 weeks post-vaccination (pv) as well as 2 weeks post-infection (pi). Annotations of the horizontal axes refer to chicken numbering. Each filled symbol represents the result for one chicken at the indicated time point. Empty symbols on chart B mean that the results are for sera collected at 2 weeks pv to replace the lacking data from the indicated 3 weeks pv. The 1.0 values, marked with filled symbols on charts C and D, indicate that the HI antibody titers were, if any, lower than the detection limit (1:8). Signs at the horizontal axes denote that determinations were not performed due to (x) serum loss or (+) chicken death. The bars represent the (A-B, E-F) arithmetic or (C-D) geometric means of the results from the respective tests, calculated for each vaccine group at individual time points. Geometric mean titers (GMT) of HI antibodies were obtained considering only HI-positive sera (HI titers ≥1:16). The dashed lines indicate cut-off values in the respective tests.

Vaccinated, contact and control chickens were evaluated for the presence of serum antibodies to influenza A virus antigens during the time-course of the challenge experiments. All of the positive controls tested negative in the respective tests before infection and were not examined after challenge due to death. The humoral responses in the remaining groups of chickens at 2 and/or 3 weeks post-vaccination as well as at 2 weeks post-infection in the homologous (Exp 1) and heterologous (Exp 2) challenge experiments are presented in Fig 2A–2F.

To monitor the exposure of chickens to AIVs, the AI MultiS-Screen antibody ELISA kit (Idexx Lab) was used. This test is designed to detect antibodies directed towards the antigenically conserved nucleoprotein (NP) of influenza A viruses in bird sera. The results obtained for individual chickens are shown in Fig 2A and 2B as an S/N ratio in relation to the cut-off value. Before inoculation with the challenge viruses, all of the vaccinated and contact chickens were negative in the test. This confirms that the chickens under study have not been exposed to AIVs prior to these experiments, as expected for SPF animals. In the seventeen vaccinated chickens and two contact animals that survived infections with H5N1 HPAIVs, all of the vaccinated and one contact chicken in Exp 1 as well as four vaccinated animals in Exp 2 responded to challenges by producing anti-NP antibodies. The other three vaccinated survivors of Exp 2 and the second contact chicken of Exp 1 did not develop significant antibody responses against this internal viral protein and could still be classified as negative by the test.

To evaluate the immune responses of chickens to vaccinations and challenges with H5N1 HPAIVs, the anti-H5 HA antibodies in sera were measured. This was accomplished using the HI and ID Screen Influenza H5 Antibody Competition (FluAC H5, IDVet) tests. In the HI test, which detects antibodies that block virus binding to cellular receptors, SPF chicken erythrocytes were used. Only results of the HI test with homologous H5N1 HPAIV (Table 1) could be obtained. These are presented in Fig 2C and 2D as HI antibody titers in relation to the cut-off value. Two and three weeks after the 2-dose vaccination with adjuvanted rH5-*E*. *coli*, five out of ten chickens in Exp 1 were HI positive, with titers ranging from 1:16 to 1:128 and a Geometric Mean Titer (GMT) value of 1:37 (Fig 2C). At the same time, both of the contact chickens were negative in this test. Upon infection with homologous H5N1 HPAIV, all of the vaccinated chickens and one contact bird became HI positive. The HI titers in the immunized group reached 1:128–1:512, and the GMT increased to 1:256. All of the vaccinated chickens of Exp 2 were HI positive, in contrast to the HI-negative contact birds (Fig 2D). HI titers of 1:16–1:256 and a GMT value of 1:47 were measured in these animals. Two weeks after infection with heterologous H5N1 HPAIV, HI titers between 1:16 and 1:2048 and a GMT value of 1:232 were measured in seven vaccinated survivors.

FluAC H5 is a diagnostic kit designed to specifically detect antibodies directed against the H5 antigen of influenza A viruses in bird sera. The results obtained for individual chickens are shown in Fig 2E and 2F as a competition percentage in relation to the cut-off values for positivity and negativity. At the 3-week post-vaccination time point in Exp 1, all of the vaccinated chickens were positive and the contact birds negative (Fig 2E). After infection with homologous H5N1 HPAIV, the vaccinated chickens remained seropositive, and one out of two contact birds became seropositive. Subsequently to the prime-boost immunizations in Exp 2, the determined competition values varied greatly, indicating six positive and four negative chickens (Fig 2F). The samples that were collected in parallel from both contact chickens in this experiment were seronegative. Two weeks post-infection with heterologous H5N1 HPAIV, six vaccinated survivors were positive and one unknown in the FluAC H5 test.

### Vaccine-induced antibodies and protection against challenge

To determine the relationship between the humoral responses of chickens to vaccination and their survival in challenge experiments, a detailed analysis of the anti-H5 antibody profiles in sera prior to animal infections (Fig 2C–2F) was performed. The first finding was that prime-boost vaccination with adjuvanted rH5-*E*. *coli* elicited in the chicken antibodies that were active in the HI assay with homologous H5N1 HPAIV and/or in the FluAC H5 test. This result is consistent with the pre-determined biochemical characteristics of our bacterial HA (Fig B and Table B in S1 Appendix). The titers of the evoked HI antibodies were considered protective (≥1:16) and desired (≥1:64) for preventing an AIV infection (Fig 2C and 2D). In addition, the H5-subtype-specific antibodies could reach the levels observed during infection with relevant AIVs, such as H5N1 viruses (Fig 2E and 2F). Chickens that were subjected to studies in the homologous (Exp 1) and heterologous (Exp 2) challenge experiments responded differently to vaccination. In Exp 1, weaker HI but stronger H5-subtype antibody responses were observed than in Exp 2. Consistently, the positivity rates in the group of chickens numbered 1–1 to 1–10 compared to those in the chickens numbered 2–1 to 2–10 were 50% *vs*. 100% and 100% *vs*. 60% as determined by HI and FluAC H5 tests, respectively. The differences between rH5-*E*. *coli* preparations, which went unnoticed in the tests applied for antigen characterization, cannot be excluded as a possible cause of the observed response divergence.

The second finding relates the vaccine-induced antibodies against H5 HA in individual chickens to their survival from viral infection. The relevant results are presented in Figs 2C–2F and 1A and 1B. For clarity, the competition percentage values, determined in sera after prime-boost immunizations with the FluAC H5 test, were placed on the chart in ascending order corresponding to the chicken numbering (Fig 2E and 2F). The same chicken numbering was preserved in Fig 2B and 2C, showing HI antibody titers. Five chickens of Exp 1 (1–1 to 1–4, 1–9), classified as positive in the HI and FluAC H5 tests after vaccination, as well as the remaining birds in the group (1–5 to 1–8, 1–10), being HI negative but FluAC H5 positive, survived infection with the homologous H5N1 HPAIV (Figs 2C, 2E and 1A, respectively). This suggests that H5-subtype-specific antibodies, detected by the FluAC H5 test, may be sufficient to confer resistance to H5N1 virus infection. Six chickens of Exp 2 (2–1 to 2–6), both HI and FluAC H5 positive after vaccination, and one animal (2–7), HI positive and FluAC H5 negative, survived challenge with heterologous H5N1 HPAIV (Figs 2D, 2F and 1B, respectively). In contrast, the other three birds (2–8 to 2–10), determined to be HI positive but FluAC H5 negative, died upon infection. Apparently, low levels of antibodies inhibiting hemagglutination by homologous H5N1 HPAIV of unknown cross-clade reactivity were not protective. The results of Exp 2 again suggest the role of H5-subtype-specific antibodies, which were active in the FluAC H5 test, in conferring anti-influenza virus immunity. Nevertheless, HI antibodies reacting with homologous (Exp 1) or heterologous (Exp 2) H5N1 viruses could also contribute to the observed protection.

### Replication, shedding and transmission of challenge viruses

**Fig 3:**
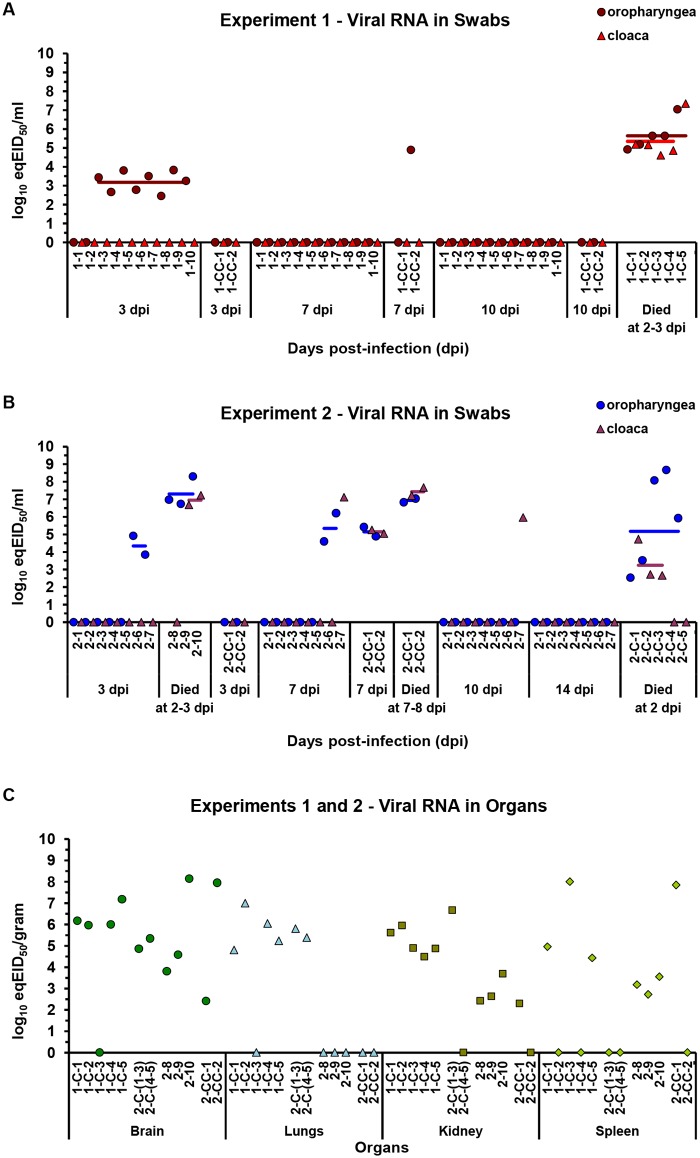
Viral RNA detection after challenging the chickens. The chickens were vaccinated and challenged as described in Fig 1. The amount of viral RNA in the (A-B) swabs and (C) organs of chickens after infection with (A, C) homologous (Exp 1) and (B, C) heterologous (Exp 2) H5N1 HPAIVs was determined by quantitative real-time mRT-PCR. Analyses were conducted as described in the Materials and Methods. Viral RNA titers were expressed as log_10_ eqEID_50_ (50% egg infectious dose) *per* milliliter of swabs or gram of tissue. The results for the vaccinated, contact and control chickens in Exp 1 and Exp 2 are presented. For animals that survived the (A) homologous or (B) heterologous challenge, data on viral RNA in oropharyngeal and cloacal swabs collected at (A) 3, 7 and 10 (Exp 1) or (B) 3, 7, 10 and 14 (Exp 2) days post-infection (dpi) are provided. The data are completed with results from analyses of (A-B) swab samples and (C) brains, lungs, kidneys and spleens collected postmortem (pm) from chickens that died upon infection at the indicated number of days post-infection. Filled symbols, differentiated between (A-B) oropharyngeal and cloacal swabs or (C) organs, represent the results for individual or pooled samples collected from chickens as indicated by the numbers on the horizontal axes. A value of zero indicates that viral RNA was not detected. Bars represent the Geometric Mean Titers (GMT) of viral RNA in (A, B) oropharyngeal or cloacal swabs and (C) organs collected from individual chicken groups at the subsequent number of days post-infection and/or postmortem. Only virus-positive samples were considered in the GMT calculations.

The vaccinated, contact and control chickens in Exp 1 and Exp 2 were evaluated for the presence of homologous and heterologous H5N1 HPAIVs following challenges with the respective viruses. The evaluation was based on the viral RNA determinations in oropharyngeal and cloacal swabs collected at 3, 7 and 10 dpi in Exp 1 and at 3, 7, 10 and 14 dpi or postmortem (pm) in Exp 2. Testing of swabs was completed with analyses of organs from birds that died upon infection. The samples were examined with quantitative real-time mRT-PCR. The results were expressed as log_10_ eqEID_50_ *per* milliliter of swabs or gram of tissue and are presented in Fig 3A–3C.

Among the ten chickens that were vaccinated in Exp 1 and then infected with homologous H5N1 HPAIV, eight were found to be virus positive (Fig 3A). Viral RNA was detected at 3 dpi in oropharyngeal swabs, wherein the levels thereof reached up to 3.8 log_10_ eqEID_50_/mL. At the subsequent number of days post-infection, the virus was detectable neither in the animal’s oropharyngea nor in the cloaca. On day 7 after the vaccinated birds had been challenged, viral RNA was detected in an oropharyngeal swab from one out of two contact chickens and thereafter was no longer detected. The second contact chicken was free of the challenge virus. Altogether, the results of Exp 1 indicate that the two vaccinated chickens did not shed the virus and that the remaining eight animals of the test group shed the virus only transiently (maximally up to 7 days). Importantly, effective virus transmission to fully susceptible contact chickens did not occur, resulting in animal survival (Fig 1A).

Among the seven vaccinated chickens of Exp 2 that survived the challenge with heterologous H5N1 HPAIV, only two were virus positive (Fig 3B). In one of these chickens, denoted 2–6, viral RNA was detected in oropharyngeal swabs at 3 and 7 dpi and in the second bird, denoted 2–7, in oropharyngeal and/or cloacal swabs at 3, 7 and 10 dpi. The highest RNA titer in chicken 2–6 was measured at 3 dpi (4.9 log_10_ eqEID_50_/mL), while in chicken 2–7, the highest titer was measured at 7 dpi in both oropharyngeal (6.2 log_10_ eqEID_50_/mL) and cloacal (7.1 log_10_ eqEID_50_/mL) swabs. Notably, the latter chicken with the extremely high virus level was the only animal surviving infection with H5N1 HPAIVs that did not develop antibodies active in the FluAC H5 test (Fig 2E and 2F). At 10 dpi and 14 dpi, six and all of the survivors of the heterologous challenge, respectively, had no detectable H5N1 virus. In the three vaccinated chickens that died at 2 or 3 dpi, substantial amounts of heterologous viral RNA were determined pm in swabs from oropharyngea and/or cloaca, at 6.7–8.3 log_10_ eqEID_50_/mL (three birds) and 6.7–7.2 log_10_ eqEID_50_/mL (two birds), respectively. Between 3 and 7 dpi, the challenge virus was transmitted to both contact chickens. At 7 dpi, large amounts of heterologous viral RNA were detected in the swabs of these animals. Even greater virus titers were determined pm in samples from the cloaca (6.8 and 7.0 log_10_ eqEID_50_/mL) and oropharyngea (7.2 and 7.7 log_10_ eqEID_50_/mL) of the birds found dead from 7 to 8 dpi. To summarize, the results of Exp 2 showed that five chickens did not and that two others only transiently (maximally up to 10 or 14 days) shed the virus of a total of ten vaccinated animals. Although delayed, the effective transmission of the heterologous virus to fully susceptible contact animals occurred, resulting in animal deaths (Fig 1B).

In the non-vaccinated and infected chickens, which were the control groups for the challenge experiments, large amounts of the virus were found after the birds’ death at 2 or 3 dpi (Fig 3A and 3B). The virus titers reached values up to 7.0 (Exp 1) or 8.7 (Exp 2) log_10_ eqEID_50_/mL in oropharyngeal and up to 7.3 (Exp 1) or 4.7 (Exp 2) log_10_ eqEID_50_/mL in cloacal swabs. This indicates the strong multiplication of H5N1 HPAIVs in the non-vaccinated chickens. By comparison, the lack in seven and the significantly reduced viral replication in the vast majority of the remaining vaccinated survivors were noted throughout the observation period (Fig 3A and 3B). In nine out of ten chickens with detected virus replication, the H5N1 viruses were recovered only from the oropharyngea. Independently of the location and peak levels, H5N1 viruses were completely cleared from all of the virus-positive chickens.

The results of organ analyses by PCR are shown in Fig 3C. As observed, substantial amounts of viral RNA were detected in most or all of the organs from chickens that died upon infection, namely, five controls in Exp 1 (Fig 1A) and two contact, three vaccinated and five control birds in Exp 2 (Fig 1B). The determined viral RNA titers in the brain, lung, kidney and spleen samples amounted to 8.1, 7.0, 6.7 and 8.0 log_10_ eqEID_50_ *per* gram of tissue, respectively. Noteworthy, no virus was detected in the lungs of dead vaccinated and contact chickens from Exp 2. Nevertheless, the pronounced systemic dissemination of greatly multiplied H5N1 viruses in all of the chickens took place before their death.

## Discussion

H5N1 HPAIVs remain a serious epidemiological problem. Emerging disease outbreaks are accompanied by high virulence and mortality among birds, and bird-to-human transmission causes severe disease with a frequently fatal outcome. Moreover, these viruses may acquire the ability of human-to-human transmission and thereby pose a pandemic threat. To control H5N1 HPAIVs, HA-based vaccines are being developed using predominantly eukaryotic expression systems (e.g., [8–10]). More recently, so-called bacterial HAs have become a promising alternative to vaccine antigens expressed in yeast, baculovirus-insect or mammalian cells. After the failure to obtain bacterial HA with native protein characteristics in the early 1980s, an increasing amount of data have been published from 2007 on the successful production of different subtype HA antigens in bacteria (for review, see [11]). The majority of these antigens comprised sequences derived solely from the HA1 subunit. Only a few reports on ectodomain-based bacterial HAs for subtype-specific vaccine production have been presented so far. They include the 60-kDa [16] and 63-kDa [17] H5 HAs, the 97-kDa msyB:H5 protein [18] and the 50-kDa H1pdm09 HA [19].

The ectodomain-based H5 HA protein presented here has never before been produced bacterially or described as an effective antigen for vaccination against influenza, including that evoked by H5N1 HPAIVs. The antigen, referred to as rH5-*E*. *coli*, has several unique features. It does not contain a signal peptide, which is also absent from the mature HA protein. The protein is truncated to remove immunologically inert protein regions, including the transmembrane and cytoplasmic domains. Moreover, the C-terminal fragment downstream of the bromelain cleavage site was also removed. Thus, rH5-*E*. *coli* is clearly distinguishable from the full-length ectodomain-based H5 HA [16] and the 1-480-aa fragment of the H1pdm09 HA [19]. In the rH5-*E*. *coli*, the basic amino acids from the cleavage site between the HA1 and HA2 subunits of the source HA sequence (Table 1) were deleted (ΔRRRKKR). This is in contrast to the previously reported ectodomain-based bacterial H5 HA with the polybasic amino acid region intentionally included [16]. This site is the main determinant of the high pathogenicity of H5N1 AIVs [20]. For safety reasons, it is absent from the HP viral strains of the conventional influenza vaccines. Although this is not the case of the subunit vaccine, the region susceptible to protease cleavage could introduce instability to the H5 HA protein under production.

rH5-*E*. *coli* retains immunologically important regions of naturally occurring influenza virus HAs. It contains the sequence of the highly variable HA1 subunit that, in the native HA, forms immunodominant and subtype-, clade- and strain-specific neutralizing epitopes (for review, see [21]). This antigen also comprises the sequence of the HA2 subunit fragment, which is relatively well conserved and in viral HA contains hetero-subtype-neutralizing epitopes [22–25]. Therefore, our approach is more advantageous than the HA1 subunit-based designs. The native HA fragment, corresponding to rH5-*E*. *coli*, encompasses epitopes targeted by antibodies neutralizing influenza virus infectivity *via* various mechanisms [21,26]. Thus, anti-HA1 subunit antibodies could act mainly by blocking the binding of the viral HA to cellular receptors, and anti-HA2 subunit antibodies could do so by interfering with the subsequent HA-mediated membrane fusion.

The prototype vaccine H5 HA (aa 17–522, ΔRRRKKR) with the sequence from the H5N1 HP viral strain (Table 1) was efficiently expressed in *E*. *coli* due to the removal of the native HA hydrophobic regions (the signal peptide and the transmembrane domain) and to codon optimization. Because the target antigen was expressed in the form of IBs, the main challenge of our bacterial HA production was to develop a refolding method that would obtain a similar antigen to viral HA. The lack of a signal sequence and an anchoring peptide participating in HA folding during biosynthesis and the truncation of the HA2 subunit involved in native HA trimer formation [27] were potential difficulties. According to the final procedure, the target protein was refolded by dilution between two chromatographic purification steps. The value of the final rH5-*E*. *coli* was verified by studies on its antigenicity, ability to bind to sialic acid-containing receptors and oligomerization status. These studies showed that rH5-*E-coli* displays well-preserved conformational epitopes targeted by H5-subtype specific, HI and VN antibodies (Fig B in S1 Appendix). Both the proper conformation of the obtained antigen and its capacity to form functional oligomers were evidenced by the hemagglutination test (Table B in S1 Appendix). The oligomerization of rH5-*E*. *coli* occurred in the absence of foreign trimerizing sequences, encountered in the recombinant, soluble HAs produced in mammalian or insect cells [10,28–31]. The process was possibly mediated by the HA2 subunit fragment. For comparison, the 1-480-aa H1pdm09 did not display oligomeric HA activities and existed as a correctly folded monomer [19]. The oligomerization status of the other ectodomain-based bacterial HAs has not been described. The oligomeric nature of rH5-*E*. *coli* favorably distinguishes it from the monomeric, HA1 subunit-based bacterial HAs [32–34]. The beneficial effect of HA oligomerization on the protective efficacy, cross-protection and/or dose sparing has been clearly indicated from studies with HAs from both eukaryotic [28,30] and prokaryotic [35,36] expression systems.

Our bacterial HA presents some advantages over both the longer and the shorter bacterially produced HA proteins. In comparison to the full-length, ectodomain-based H5 HA protein [16], rH5-*E*. *coli* is characterized by decreased hydrophobicity and increased stability due to removal of the C-terminal fragment downstream of the bromelain cleavage site and the basic amino acids from the inter-subunit cleavage site, respectively. In contrast to the shorter, ectodomain-based, 1-480-aa H1pdm09 protein [19], rH5-*E*. *coli* forms functional oligomers. By its ability to oligomerize, rH5-*E*. *coli* presents a significant advantage over conformational, HA1 subunit-based bacterial HAs, which are exclusively monomeric (e.g., [32–34]). In contrast to antigens based on the globular domain of the HA1 subunit (e.g., [37–38]), production of different-origin HA proteins according to our design does not demand *in silico* modeling. It is because the sequences may be simply determined by identification of the bromelain cleavage sites. In comparison to all of the HA1 subunit-based vaccines, the presence of more conservative neutralizing epitopes of the HA2 subunit in rH5-*E*. *coli* increases its potential for cross-protection. Advantages of the HA antigen, designed by us, justify our effort to verify its value in the challenge experiments.

The capacity of rH5-*E*. *coli* to confer protection against H5N1 HPAIVs was studied in the SPF laying-type chickens. As a proof of concept for our bacterial H5 HA, a relatively high 25-μg dose of the antigen was used. rH5-*E*. *coli* was given to birds twice in the presence of widely approved and cheap aluminum hydroxide adjuvant. Vaccination resulted in a 100% survival rate in the homologous challenge experiments (Fig 1A). Full protection of chickens against H5N1 HPAIV was also achieved with the 97-kDa msyB:H5 protein [18], which as the only one among the aforementioned ectodomain-based bacterial H5 HAs, passed an efficacy challenge test. However, the result was obtained with incomparably higher doses of antigen than used in this work. Vaccination with rH5-*E*. *coli* protected 70% of chickens from high-dose challenge exposure to heterologous H5N1 HPAIV (Fig 1B). Thus, at that time, our vaccine did not fulfill the criteria established by the World Organization for Animal Health (OIE) for the licensing of conventional vaccines, which is a minimum of 80% protection from mortality in the vaccine group [13]. The problem of ensuring cross-protection even against related influenza virus strains with HA-based vaccines is widely recognized. Attempts to resolve it are focused on the design of both adjuvants and antigens. The ability to elicit broader protection was demonstrated with MF59 adjuvant applied to inactivated H5N1 vaccines [39]. The designs frequently give the HA protein the ability to form oligomeric structures. This is relevant for the titer and repertoire of the induced antibodies [28,30,40] and thus for the protection level. A clear indication for this came from studies on the efficacy of the HA1 subunit-based bacterial H5 HA performed in the ferret model [36]. In the homologous clade 1 and heterologous clade 2.2 H5N1 HPAIV challenges, 100% and 80% survival rates, respectively, were achieved with largely oligomeric HA; these rates were 100% in both cases with the oligomeric fraction of the same antigen. Because rH5-*E*. *coli* only partially exists as a functional oligomer (Table B in S1 Appendix), a solution for providing higher inter-clade protection with our bacterial HA may be augmented oligomerization.

The protection of chickens against both homologous and heterologous H5N1 HPAIV infection, provided by vaccination with adjuvanted rH5-*E*. *coli*, was primarily associated with antibodies detected by the commercial FluAC H5 test (Fig 2E and 2F). Thus, our work showed novel functional antibodies with inter-clade reactivity. It can be reasonably assumed that these antibodies recognize the subtype-specific epitope in the HA1 subunit of viral H5 HAs and do not have HI activity. They may belong to the category of anti-influenza virus antibodies with broad cross-clade neutralizing activities that have already been identified among mAbs against HA1 subunit of HA from H5N1 viruses (for review, see [21]). The exemplary antibody clones, designated 9F4 and HA-7, were demonstrated to inhibit viral entry not at the receptor-binding step but during post-attachment events [41,42]. The mechanism of neutralization shown for mAb 9F4 relies on blocking the low pH-induced conformational change of HA, which is prerequisite for membrane fusion and subsequent viral RNA release into the host cell [41]. As indicated by the 100% FluAC H5-positivity in the test vaccine group of chickens from Exp 1 (Fig 2E), the induction of newly established functional antibodies with rH5-*E*. *coli* in all vaccinated animals is possible. It can be further speculated that obtaining such a result in the chickens of Exp 2 (instead of 60% FluAC H5-positivity) (Fig 2F) could lead to the full protection of birds against challenge with heterologous H5N1 HPAIV.

The immunity of individual chickens against H5N1 viruses achieved in this work was weakly, if at all, related to the induction of HI antibodies. Although the level of HI antibody titer is widely considered as the correlate of vaccine-induced protection [43], protection against challenge in the absence of detectable HI antibodies has already been reported. Examples are provided from studies on flagellin-H5 HA [44] and whole-H5 virus [45] vaccines in ferrets, H5N1 VLPs in ferrets [46] and mice [47] and DNA vaccine encoding H5 HA in chickens [48]. These and the findings presented here note that the results of the HI test may be misleading, especially if newly designed HA antigens are tested. In contrast, positivity in the FluAC H5 test indicated to us predictive value for the protective efficacy of vaccines against H5N1 HPAIVs. This test might successfully be used in the evaluation of the vaccine potential of H5 HA proteins under development. On the other hand, the exploitation of the H5 subtype-conserved epitope targeted by antibodies competing in the FluAC H5 test would be useful for the design of pre-pandemic vaccines against circulating H5N1 viruses and perhaps other H5-subtype HPAIVs, such as the novel H5N8 and H5N2 viruses.

Protective immune responses induced by vaccination with rH5-*E*. *coli* greatly prevented viral replication in 41% of survivors and contributed to the complete clearance of replicating viruses from respiratory and digestive tracts in 59% of survivors (Fig 3A and 3B). This means that total of 85% chickens surviving challenges with H5N1 HPAIVs did not or only transiently shed the viruses. Considering all of the vaccinated birds, no chickens shed homologous virus at 7 dpi, and the number of chickens shedding heterologous virus was reduced by 70% at 14 dpi. As a consequence of complete chicken protection against homologous H5N1 HPAIV challenge, which was accompanied by the lack of or greatly reduced and transient viral replication, effective virus transmission to fully susceptible contact birds did not occur, resulting in animal survival (Fig 1A). Probably due to different patterns of immune responses (Fig 2C–2F), only partial protection of vaccinated chickens against heterologous H5N1 HPAIV challenge was achieved (Fig 1B). Although with a delay, this led to virus transmission and death of the contact birds. Nevertheless, our studies clearly indicate the potential of the novel bacterial H5 HA to control H5N1 HPAIV infection and transmission rates in the chicken flocks as well as to reduce viral shedding. In addition, these studies demonstrate that bacterially produced HA can be a valuable vaccine antigen when appropriate folding and purification methods are applied to the rationally designed protein. The relative ease and low-cost production of rH5-*E*. *coli* associated with bacterial expression make it a promising alternative to vaccine HAs produced at higher costs in eukaryotic expression systems. Similar to other subunit vaccines against influenza, bacterial HA can be used in preventive or emergency vaccinations in compliance with a DIVA strategy.

### Addendum

The yields and the amounts of the rH5-*E*.*coli* obtained in this work were sufficient to achieve the objectives of the present study, however they were too low to use the antigen in mass vaccinations. Therefore, the procedure was further optimized and scaled-up. As a result, a technological process for the production of designed H5 HA protein on a large scale was developed (to be described in a separate paper).

## Supporting information

S1 Appendix Fig A in S1 Appendix SDS-PAGE showing (A) expression and (B) purification of rH5-*E*. *coli*. Fig B in S1 Appendix Antigenicity of rH5-*E*. *coli*. Table A in S1 Appendix Anti-H5 hemagglutinin antibodies used in this work. Table B in S1 Appendix Hemagglutination activity of rH5-*E*. *coli*. S1 Appendix Supplementary data on vaccine antigen.
(DOC)
