# Correction: A novel hemagglutinin protein produced in bacteria protects chickens against H5N1 highly pathogenic avian influenza viruses by inducing H5 subtype-specific neutralizing antibodies

**DOI:** 10.1371/journal.pone.0176249

**Published:** 2017-04-17

**Authors:** Violetta Sączyńska, Agnieszka Romanik, Katarzyna Florys, Violetta Cecuda-Adamczewska, Małgorzata Kęsik-Brodacka, Krzysztof Śmietanka, Monika Olszewska, Katarzyna Domańska-Blicharz, Zenon Minta, Bogusław Szewczyk, Grażyna Płucienniczak, Andrzej Płucienniczak

Fig A and Fig B are omitted from the S1 Appendix file. Please see the complete, corrected S1 Appendix here.

## Supporting information

S1 Appendix Fig A in S1 Appendix SDS-PAGE showing (A) expression and (B) purification of rH5-*E*. *coli*. Fig B in S1 Appendix Antigenicity of rH5-*E*. *coli*. Table A in S1 Appendix Anti-H5 hemagglutinin antibodies used in this work. Table B in S1 Appendix Hemagglutination activity of rH5-*E*. *coli*. S1 Appendix Supplementary data on vaccine antigen.
(DOC)
