# Supplementary material for: Correction: A novel hemagglutinin protein produced in bacteria protects chickens against H5N1 highly pathogenic avian influenza viruses by inducing H5 subtype-specific neutralizing antibodies
Source: PLoS One. 2017 Apr 17;12(4):e0176249. doi: 10.1371/journal.pone.0176249 (PMC5393861; doi:10.1371/journal.pone.0176249)
Supplement: S1 Appendix — Fig A in S1 Appendix SDS-PAGE showing (A) expression and (B) purification of rH5-E. coli. Fig B in S1 Appendix Antigenicity of rH5-E. coli. Table A in S1 Appendix Anti-H5 hemagglutinin antibodies used in this work. Table B in S1 Appendix Hemagglutination activity of rH5-E. coli. S1 Appendix Supplementary data on vaccine antigen. (DOC) [file pone.0176249.s001.doc]

**S1 Appendix. Supplementary data on vaccine antigen.**

**Production of rH5-*E. coli***

The hemagglutinin (HA) protein (aa 17-522, ΔRRRKKR) with the sequence of the A/swan/Poland/305-135V08/2006(H5N1) strain of the highly pathogenic (HP) avian influenza virus (AIV) was efficiently expressed in the transformed BL21 (DE3) *Escherichia coli* cells. The expressed protein was recovered by isolation of inclusion bodies followed by solubilization of the protein under denaturing conditions. To evaluate the proof-of-concept for our bacterial H5 HA, a quality-focused procedure was used in the laboratory level production of the target antigen. According to this procedure, proteins solubilized from inclusion bodies were subjected sequentially to purification on a DEAE-Sepharose column, refolding by dilution and purification on a Phenyl-Sepharose column. At the successive stages, the following recovery efficiencies were obtained: 20-25%, 18-25% and 1.5-2.5% of the initial protein load (67-87 mg /L culture). Thus, the employed procedure comprised of a highly efficient refolding of the protein and a low yield protein purification on a Phenyl-Sepharose bed, which limited the overall process efficiency. Finally, 1-2 mg of antigen, 75-80% pure, was produced *per* liter of bacteria culture. This amount was sufficient to verify the protective efficacy of our bacterial H5 HA (rH5-*E. coli*) in the challenge experiments. The process of the rH5-*E. coli* production was further optimized and scaled-up.

**Gel electrophoresis**

The expression of the HA protein and its purification were followed by sodium dodecyl sulfate-polyacrylamide gel electrophoresis (SDS-PAGE). The samples collected after each step of antigen production were mixed with an equal volume of Laemmli sample buffer (2X) and thereafter boiled for 5 min. SDS-PAGE was performed under reducing conditions using a 3.5% stacking gel, pH 6.8 and a 15% resolving gel, pH 9.2. Low molecular weight (LMW) protein marker (94, 67, 43, 30, 20.1, and 14.4 kDa) was run along with the samples. Coomassie Brilliant Blue G-250 was used to visualize the proteins. LMW protein marker was purchased from GE Healthcare (Sweden). All other reagents that were used to perform SDS-PAGE were purchased from Sigma-Aldrich (USA). An example of SDS-PAGE analysis is given in Fig A.


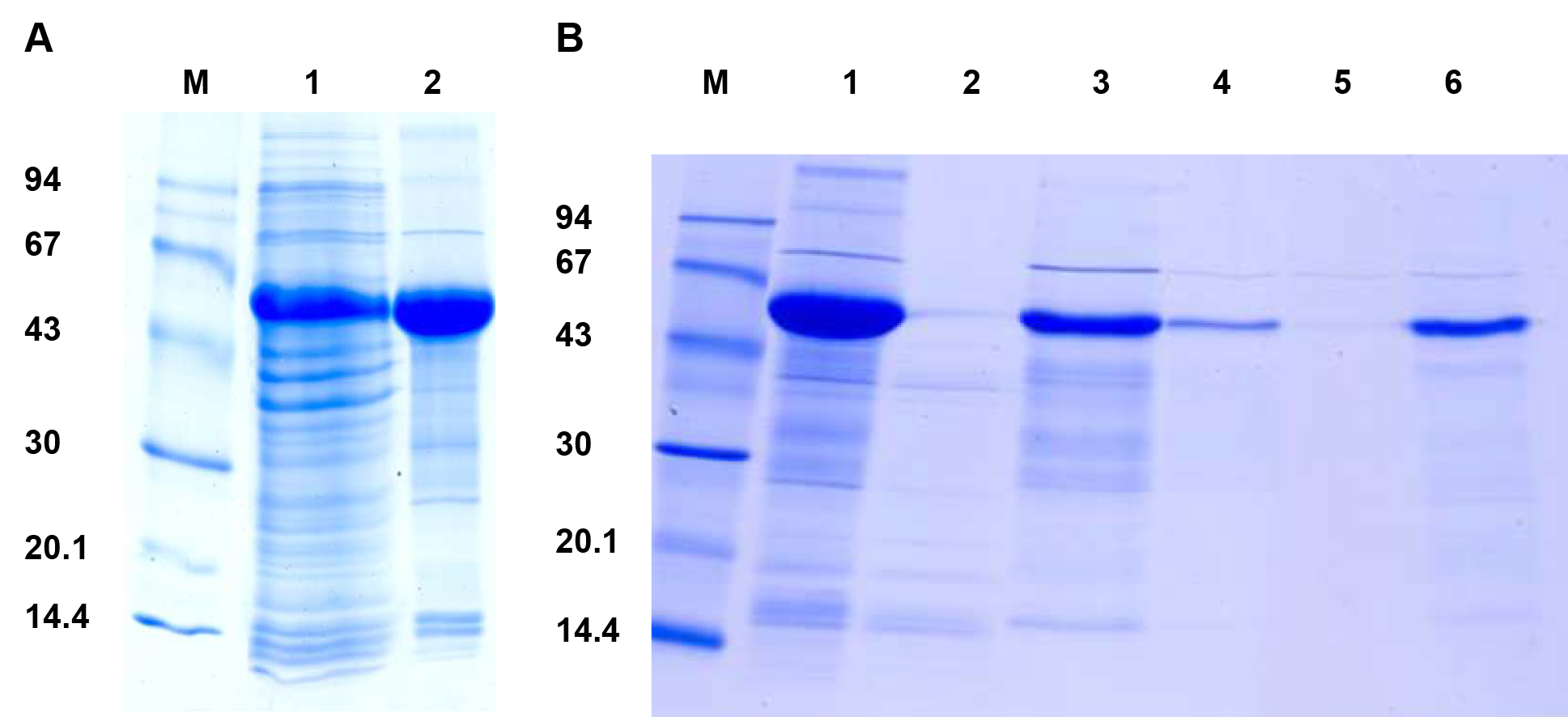


**Fig A. SDS-PAGE showing (A) expression and (B) purification of rH5-*E. coli*.** (A) M, low molecular weight protein marker (kDa); 1, *Escherichia coli* lysate; 2, isolated inclusion bodies. (B) M, low molecular weight protein marker (kDa); 1, inclusion bodies after solubilization; 2 and 3, a flow-through fraction and eluted peak fractions from a DEAE Sepharose Fast Flow column, respectively; 4, proteins after refolding; 5 and 6, a flow-through fraction and eluted peak fractions from a Phenyl Sepharose 6 Fast Flow (High-Sub) column, respectively.

**Antibodies for antigenicity studies**

In the analyses of rH5-*E. coli*, commercially available antibodies (Abs) against influenza virus H5 HA, both monoclonal (mAbs) and polyclonal (pAbs), were used. A total of 9 clones of Abs were purchased from USBiological, USA (3 clones); Thermo Scientific, USA (1 clone); and Acris Antibodies, Germany (5 clones). For their production, purified H5N1 avian influenza A viruses (8 clones) or the HA protein of A/Vietnam/1203/04(H5N1) influenza virus (1 clone) was used as immunogens. The produced mouse mAbs, all of the IgG2a isotype, are further referred to as mAb 1 to mAb 9. Most of these mAbs (6/9) were specified as reactive with the H5N1, H5N2 and H5N9 influenza viruses, non-cross-reactive with HA of H1-H4 and H6-H15 subtypes and recognizing H5 HAs in the hemagglutination inhibition (HI) test. One out of 8 clones with HI activity was also characterized as active in the virus neutralization (VN) test. The rabbit IgG antibodies were obtained using proteins with amino acid sequences 1‑345 and 347‑523 of HAs from the A/Bar-headed Goose/Qinghai/12/05(H5N1) and A/VietNam/1203/2004(H5N1) influenza viruses, respectively, for animal immunization (Immune Technology Corp., USA). To distinguish between these pAbs, antibodies directed against HA1 and HA2 subunits of antigen are further referred to as pAb 1 and pAb 2, respectively. The data provided by manufacturers on mAb 1 to mAb 9, pAb 1 and pAbs 2 are summarized in Table A.

**Table A**. Anti-H5 hemagglutinin antibodies used in this work.

| **Denotation** | **Source, Cat. No.** | **Immunogen** | **Isotype** | **Application** |
| --- | --- | --- | --- | --- |
| **Monoclonal antibodies (mAbs) against H5 hemagglutinin** | | | | |
| **Recognizes H5 HA in the HI test. Reacts with H5N1, H5N2 and H5N9 strains. Does not cross-react with other HA types (H1, H2, H3, H4, H6, H7, H8, H9, H10, H11, H12, H13, H14 and H15).** | | | | |
| **mAb 1** | USBiological, 17649-41B | Purified AIV type A (H5N1) | Ms IgG2a* | HI, iELISA, DB |
| **Recognizes the H5 HA protein of influenza A virus [strain A/Vietnam/1203/04(H5N1)]. Does not cross-react with influenza viruses of other HA subtypes. Reacts with H5N1 influenza virus representatives of different clades and subclades of the H5 subtype.** | | | | |
| **mAb 2** | USBiological, 17649-42C | HA of the A/Vietnam/1203/04(H5N1) influenza virus | Ms IgG2a* | HI, VN, ELISA, IP, IHC, WB |
| **Recognizes H5 HA in HI test.** | | | | |
| **mAb 3** | USBiological, 17649-42D | Purified AIV type A (H5N1) | Ms IgG2a* | HI, ELISA |
| **Detects influenza A H5 antigen in viral samples.** | | | | |
| **mAb 4** | ABR/Thermo Scientific, MA1-81927 | Purified AIV type A (H5N1) | Ms IgG2a* | ELISA |
| **React with H5 HA in HI test. React with H5N1, H5N2 and H5N9 strains. Do not cross-react with other HA types (H1, H2, H3, H4, H6, H7, H8, H9, H10, H11, H12, H13, H14 and H15).** | | | | |
| **mAb 5** | Acris Antibodies, AM00942PU-N | Purified AIV type A (H5N1) | Ms IgG2a* | HI, iELISA, DB |
| **mAb 6** | Acris Antibodies, AM00943PU-N | Purified AIV type A (H5N1) | Ms IgG2a* | HI, iELISA, DB |
| **mAb 7** | Acris Antibodies, AM00944PU-N | Purified AIV type A (H5N1) | Ms IgG2a* | HI, iELISA, DB |
| **mAb 8** | Acris Antibodies, AM00945PU-N | Purified AIV type A (H5N1) | Ms IgG2a* | HI, iELISA, DB |
| **mAb 9** | Acris Antibodies, AM00941PU-N | Purified AIV type A (H5N1) | Ms IgG2a* | HI, iELISA, DB |
| **Polyclonal antibodies (pAbs) against H5 hemagglutinin** | | | | |
| **Reacts with human and avian H5 (H5N1). Cross-reactivity to other HAs not tested.** | | | | |
| **pAb 1** | Immune Technology Corp., IT-003-005G | HA (aa 1-345) (H5N1) (A/Bar-headed Goose/Qinghai/12/05(H5N1)) | Rb IgG* | WB |
| **Reacts with HA and HA2 (H5N1). Cross-reactivity against other subtypes not tested.** | | | | |
| **pAb 2** | Immune Technology Corp., IT-003-010 | HA2 (H5N1) protein (aa 347-523) (A/VietNam/1203/2004(H5N1)) | Rb IgG* | WB |

Ms - Mouse; Rb – Rabbit; *purified; HI - hemagglutination inhibition test; iELISA - indirect ELISA (enzyme-linked immunosorbent assay); DB - dot blot; VN - virus neutralization test; IP - immunoprecipitation; IHC - immunohistochemistry; WB - Western blot.

The utility of the selected Abs for analyses of rH5-*E. coli* was further evaluated by testing their reactivity with commercially available H5 HA proteins. The panel of antigens comprised the His-tagged ectodomain- or HA1 subunit-based proteins, referred to as rHA and rHA1, respectively. The proteins were produced in a mammalian expression system (Immune Technology Corp., USA) except for one rHA protein of baculovirus-expression system origin (Oxford Expression Technologies Ltd., UK). They contained HA sequences of seven H5N1 and one of H5N2 viral strains. As a consequence, H5 HA antigens presented substantial antigenic diversity, as confirmed by a homology search against a source sequence of rH5-*E. coli* (Table 1). Immunoreactivity examinations were completed by ELISA using Ni-NTA strips (Qiagen, Germany) for protein coating. A test with mAbs and pAbs, performed in parallel, established that the vast majority of recombinant antigens (8/9), including 6 rHA and 2 rHA1 proteins, displayed native HA characteristics. Using only conformational antigens, it was shown that the tested mAbs differed in epitopic specificity and reactivity range against H5 HAs. In addition, broad-range specific mAbs were proven to recognize conformational epitopes in the HA1 subunit of H5 HAs. The remaining clones of Abs could bind to antigens produced in eukaryotic expression systems based on HA sequences from the A/swan/Poland/305-135V08/2006(H5N1) strain of influenza virus and highly homologous viruses. In addition, pAbs 1 and 2 differentiated between HA antigens in an only partially sequence-dependent manner.

Altogether, the data provided by manufacturers and the results of our immunoreactivity studies led to the conclusion that Abs, listed in Table A, are suitable for rH5-*E. coli* examination in terms of conformational integrity and the presence of H5 subtype-specific epitopes as well as those targeted by HI and VN antibodies. Thus, the selected Abs were proven to be appropriate for the evaluation of the potential of rH5-*E. coli* as a vaccine antigen.

**Reference hemagglutinin antigens**

Analyses of rH5-*E. coli* were performed in the presence of two reference antigens. The first one, corresponding to the full-length ectodomain of HA (aa 17-530), was produced with the cleavage site deletion (ΔRRRKKR) and 6x His-tag at the C-terminus using a baculovirus expression vector system (BEVS) (Oxford Expression Technologies Ltd., UK). The sequence of the antigen, referred to as rH5-BEVS, originated from the A/swan/Poland/305-135V08/2006(H5N1) strain of HPAIV, such as that of rH5-*E. coli* (Table 1). The same His-tagged HA fragment (aa 17-530, ΔRRRKKR) produced in a mammalian expression system with the sequence derived from A/Bar-headed Goose/Qinghai/12/05(H5N1) viral strain (Immune Technology Corp., USA) served as the second reference antigen. The antigen, referred to as rH5-mammalian, was highly homologous with both rH5-BEVS and rH5-*E. coli*. According to the manufacturer’s data, majority of the rH5-mammalian exists as a trimer**/**oligomer. Both of the reference antigens were specified as purified to at least 95%. The molecular masses of rH5-BEVS and rH5-mammalian were calculated from amino acid compositions using GPMAW 8.2 software (Lighthouse, Denmark) and determined using a Matrix-Assisted Laser Desorption Ionization Time of Flight (MALDI-TOF/TOF) MS instrument (4800 Plus, AB SCIEX, USA).

**ELISA for antigenicity examination**

MediSorp plates (Nunc, Denmark) were coated with rH5-*E. coli* and reference HA antigens (rH5-mammalian, rH5-BEVS) at 5 µg/mL in PBS overnight at 2-8°C. The remaining binding sites on the plates were blocked using 10% FBS/PBS. Thereafter, mAbs and pAbs (Table A), pre-diluted in 2% BSA/PBS to 1 µg/mL and 10 µg/mL, respectively, were added, and the plates were incubated overnight at 2-8°C. The antigen-bound mAbs and pAbs were detected using HRP-labeled anti-mouse IgG (γ-chain specific) pAbs and HRP-labeled anti-rabbit IgG (γ-chain specific) mAbs, respectively (both from Sigma-Aldrich, USA). Secondary Abs, diluted 1:1000 in 2% BSA/PBS (anti-mouse pAbs) or 1% BSA/PBST (anti-rabbit mAbs), were incubated on the plates for 1 hour at 37°C. The reactions were developed with TMB (Sigma-Aldrich, USA) at room temperature for 30 min and subsequently stopped by the addition of a 0.5 M H_2_SO_4_ solution. The absorption was read at 450 nm using a Synergy HT multi-detection microplate reader (BioTek Instr. Inc., USA).

The test discriminated between properly folded and misfolded HA antigens. It was successfully used to follow the properties of bacterial HAs during the development of the refolding and purification procedure. Subsequently, the same test was used for the characterizations of rH5-*E. coli*, obtained by the final protocol described in the Materials and Methods. The results of the test for rH5-*E. coli* use in the anti-H5N1 HPAIV vaccine and reference HA proteins are presented in Fig B.


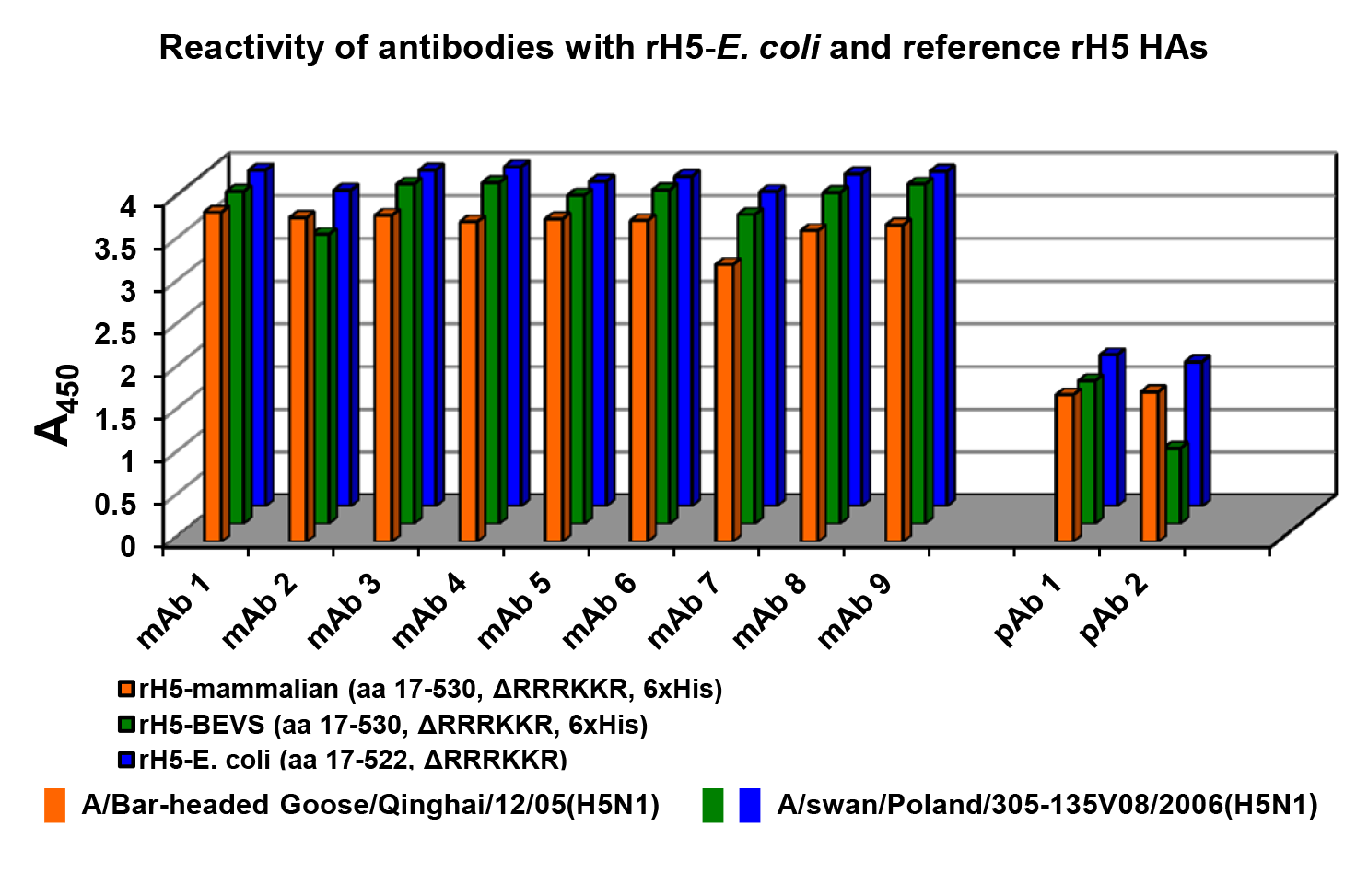


**Fig B. Antigenicity of rH5-*E. coli*.**

**Assay of hemagglutination activity**

The test was performed in V-bottom 96-well plates (CellStar/Greiner Bio-One, Germany) using red blood cells (RBCs) from specific pathogen-free (SPF) chickens (National Veterinary Research Institute, Pulawy, Poland). rH5-*E. coli* was tested in parallel with the reference antigens: rH5-BEVS and rH5-mammalian. rH5-*E. coli* reduced with 30 mM 2-mercaptoethanol, and the final buffers of each tested HA protein (50 mM Tris-HCl, pH 8.0; 50 mM Tris-HCl, pH 8.0, with 30 mM 2-mercaptoethanol; and PBS) were the negative controls. The low pathogenic A/turk/It/80(H5N2) strain of AIV, certified by Istituto Zooprofilattico Sperimentale delle Venezie (Italy) and originating from x-OvO Ltd (UK), served as a positive control. On each test plate, the erythrocyte control was included.

The rH5-*E. coli* and reference HA proteins were diluted in Dulbecco’s PBS (DPBS, Sigma-Aldrich, USA) to obtain 0.1 to 10 μg of antigens in a final volume of 100 µL. Formulation buffers were diluted similarly as were the respective proteins. The H5N2 virus samples were prepared by two-fold serial dilution in DPBS. To wells for the erythrocyte control, only the dilution buffer was added. Thereafter, 50 μL of 1% chicken RBC suspension in DPBS was added *per* well and mixed gently. Agglutination was read after incubation for 45 min at room temperature. The results of the hemagglutination test for rH5-*E. coli* and reference HA proteins are presented in Table B.

**Table B. Hemagglutination activity of rH5-*E. coli***.

| **Protein name** | **Hemagglutinin amount** | | | | | | |
| --- | --- | --- | --- | --- | --- | --- | --- |
|  | **10 μg** | **5 μg** | **2.5 μg** | **1 μg** | **0.5 μg** | **0.25 μg** | **0.1 μg** |
| **rH5-*E. coli* (aa 17-522, ΔRRRKKR)** | **+** | **+** | **+** | **+** | **+** | **-** | **-** |
| **rH5-*E. coli*, reduced** | **-** | **-** | **-** | **-** | **-** | **-** | **-** |
| **rH5-BEVS (aa 17-530, ΔRRRKKR, 6x His)** | **+** | **+** | **+** | **+/-** | **-** | **-** | **-** |
| **rH5-mammalian (aa 17-530, ΔRRRKKR, 6x His)** | **+** | **+** | **+** | **+** | **+** | **+** | **+** |
